# DNA Replication Is Intrinsically Hindered in Terminally Differentiated Myotubes

**DOI:** 10.1371/journal.pone.0011559

**Published:** 2010-07-13

**Authors:** Deborah Pajalunga, Eleonora M. R. Puggioni, Alessia Mazzola, Valentina Leva, Alessandra Montecucco, Marco Crescenzi

**Affiliations:** 1 Department of Cell Biology and Neurosciences, National Institute of Health, Rome, Italy; 2 Institute of Molecular Genetics, National Research Council, Pavia, Italy; Roswell Park Cancer Institute, United States of America

## Abstract

**Background:**

Terminally differentiated (TD) cells permanently exit the mitotic cycle while acquiring specialized characteristics. Although TD cells can be forced to reenter the cell cycle by different means, they cannot be made to stably proliferate, as attempts to induce their replication constantly result in cell death or indefinite growth arrest. There is currently no biological explanation for this failure.

**Principal Findings:**

Here we show that TD mouse myotubes, reactivated by depletion of the p21 and p27 cell cycle inhibitors, are unable to complete DNA replication and sustain heavy DNA damage, which triggers apoptosis or results in mitotic catastrophe. In striking contrast, quiescent, non-TD fibroblasts and myoblasts, reactivated in the same way, fully replicate their DNA, do not suffer DNA damage, and proliferate even in the absence of growth factors. Similar results are obtained when myotubes and fibroblasts are reactivated by forced expression of E1A or cyclin D1 and cdk4.

**Conclusions:**

We conclude that the inability of myotubes to complete DNA replication must be ascribed to peculiar features inherent in their TD state, rather than to the reactivation method. On reviewing the literature concerning reactivation of other TD cell types, we propose that similar mechanisms underlie the general inability of all kinds of TD cells to proliferate in response to otherwise mitogenic stimuli. These results define an unexpected basis for the well known incompetence of mammalian postmitotic cells to proliferate. Furthermore, this trait might contribute to explain the inability of these cells to play a role in tissue repair, unlike their counterparts in extensively regenerating species.

## Introduction

Our persistent inability to induce proliferation of terminally differentiated (TD) cells has no definite biological basis. Though perhaps superseded by more urgent questions in recent years, this problem remains unsolved.

In vertebrate species capable of extensive regeneration, such as salamanders, many cell types homologous to mammalian TD cells are not postmitotic and can proliferate in response to injuries to reconstitute organ integrity [1], [2]. Determining why our TD cells are unable to accomplish the same feat would shed light on the reasons for our poor regenerating competence.

Terminal differentiation is defined by the permanent exit from the cell cycle that takes place in the course of acquiring functional specialization. TD cells, once thought to be utterly incapable of reentering the cell cycle, have been shown to possess a largely functional proliferation machinery that can be reactivated by a number of experimental manipulations. Various types of TD cells have been reactivated by exploiting oncogenic viruses such as polyomavirus [3], SV40 [4], [5], [6], adenovirus [7], or papillomavirus [8]. Viral oncogenes are not unique in their ability to reactivate TD cells. Indeed, TD skeletal muscle cells (myotubes) can be induced to reenter the cell cycle by overexpressing cyclin D1 and cdk4/6 [9], knocking down cyclin-dependent kinase inhibitor(s) (CKIs) [10], or expressing the Twist bHLH protein [11]. TD, adult cardiomyocytes can be reactivated by forcibly expressing E2F1 [12], a variant cyclin D1 bearing a nuclear localization signal along with cdk4 [13], or the Notch2 intracellular domain [14], or by suppressing specific CKIs (F. Martelli, personal communication). Neurons synthesize DNA upon ectopic expression of E2F factors [15] and inner-ear hair cells do so following expression of the HPV E7 oncoprotein [16]. Most of these manipulations attain TD cell reactivation, at least in part, by switching on the G_1_ cyclin/cdk-pRb-E2F axis [9], [10], [12], [17], [18], an exception being possibly represented by Twist, whose mechanism of action is still under investigation.

Despite the successes in reactivating TD cells and elucidating the mechanisms thereof, inducing proliferation of TD cells remains unfeasible. To the best of our knowledge, no means to induce stable proliferation of any TD cell type has been reported, a few claims to the contrary notwithstanding. Some TD cell types can indeed attempt or undergo cell division upon forced reentry into the cell cycle. These include at least myotubes [9], [11], [19], adipocytes [9], and inner-ear hair cells [16]. However, full divisions are generally not frequent and the newly formed cells do not survive for long. Not all reports on TD cell reactivation spell out clearly the eventual fate of the cells themselves. When such fate is described, depending on the TD cell type subjected to reactivation and the means used to induce it, two outcomes are possible. In most cases, the reactivated cells undergo immediate or delayed cell death. In some instances, however, the cells arrest indefinitely in the G_2_ phase of the first cell cycle. Examples of the first type are myotubes reactivated by the adenoviral oncogene E1A [9] or the SV40 Large T antigen (TAg) [20], or by suppression of CKIs [10] and neurons forced into the cell cycle by expression of TAg [6]. Inner-ear hair cells reactivated by HPV E7 expression or by the lack of CKIs also die more or less promptly, before or after undergoing mitosis [16].

G_2_ phase arrest occurs in myotubes reactivated by overexpression of cyclin D and cdk4/6 and cardiomyocytes triggered to reenter the cell cycle by expression of E1A or E2F1 [12] or by Notch signaling [14].

In an attempt to identify the reasons for the general failure of TD cells to proliferate, we have explored the consequences of acutely suppressing specific CKIs in myotubes. We found that TD myotubes reactivated by this and other means cannot complete DNA replication, incur lethal DNA damage at the very beginning of S phase, and die by apoptosis. Since non-TD cells reactivated in the same ways suffer no detectable harm, we conclude that reactivation brings about incomplete DNA replication and DNA damage as the results of a peculiar feature of myotubes and—we propose—of most TD cells.

## Results

### Cell cycle reactivation causes mitotic aberrations and death in TD myotubes but not in quiescent fibroblasts

**Figure 1:**
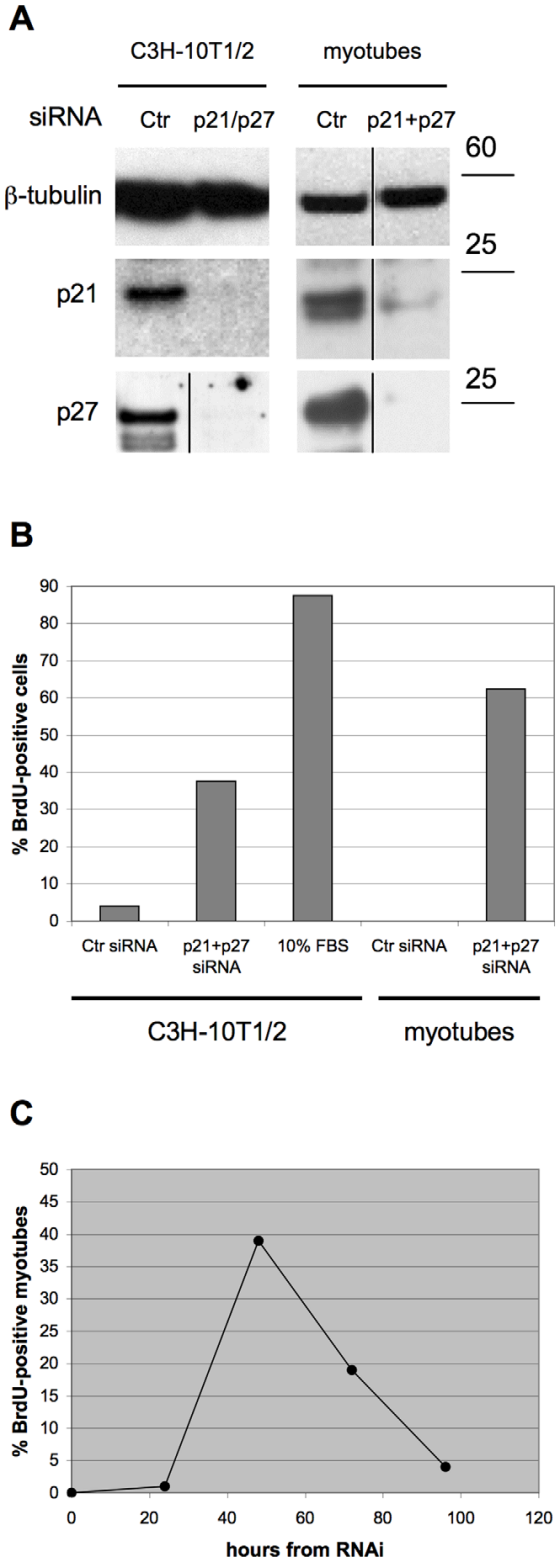
BrdU incorporation and death in cells reactivated by CKI KD. (A) quiescent C3H-10T1/2 and myotubes were transfected with the indicated siRNAs. The indicated proteins were analyzed by western blotting 48 or 20 hours later, respectively. β-tubulin is a loading control. (**B**) Serum-starved, quiescent C3H-10T1/2 fibroblasts and MSC-derived myotubes were treated as indicated and incubated with BrdU for the next 44 hours. BrdU incorporation was detected by indirect immunofluorescence. (**C**) Myotubes were transfected with a control siRNA (to GFP) or siRNAs to p21 and p27. The number of BrdU-positive myotubes (BrdU, myosin double-positive cells) was counted in 10 random, low-power microscopic fields at the indicated times post-transfection. Ctr  =  control.

To study in depth the consequences of cell cycle reactivation in TD vs. non-TD cells, we subjected TD myotubes derived from mouse satellite cells (MSC) and serum-deprived, quiescent C3H-10T1/2 mouse fibroblasts to RNA interference (RNAi) for p21 and p27 (Fig. 1A). As previously reported, interference with relevant CKIs induces reentry into the cell cycle of quiescent, senescent, and TD cells alike [10]. Fig. 1B shows that 37% of the fibroblasts and 62% of the myotubes incorporated 5-bromo-2′-deoxyuridine (BrdU) in these conditions. However, the eventual fates of the two cell types diverge strikingly. While the fibroblasts, as already shown, proliferate in the following days in the continuing absence of serum [10], the reactivated myotubes die within 3 days (Fig. 1C).

**Figure 2:**
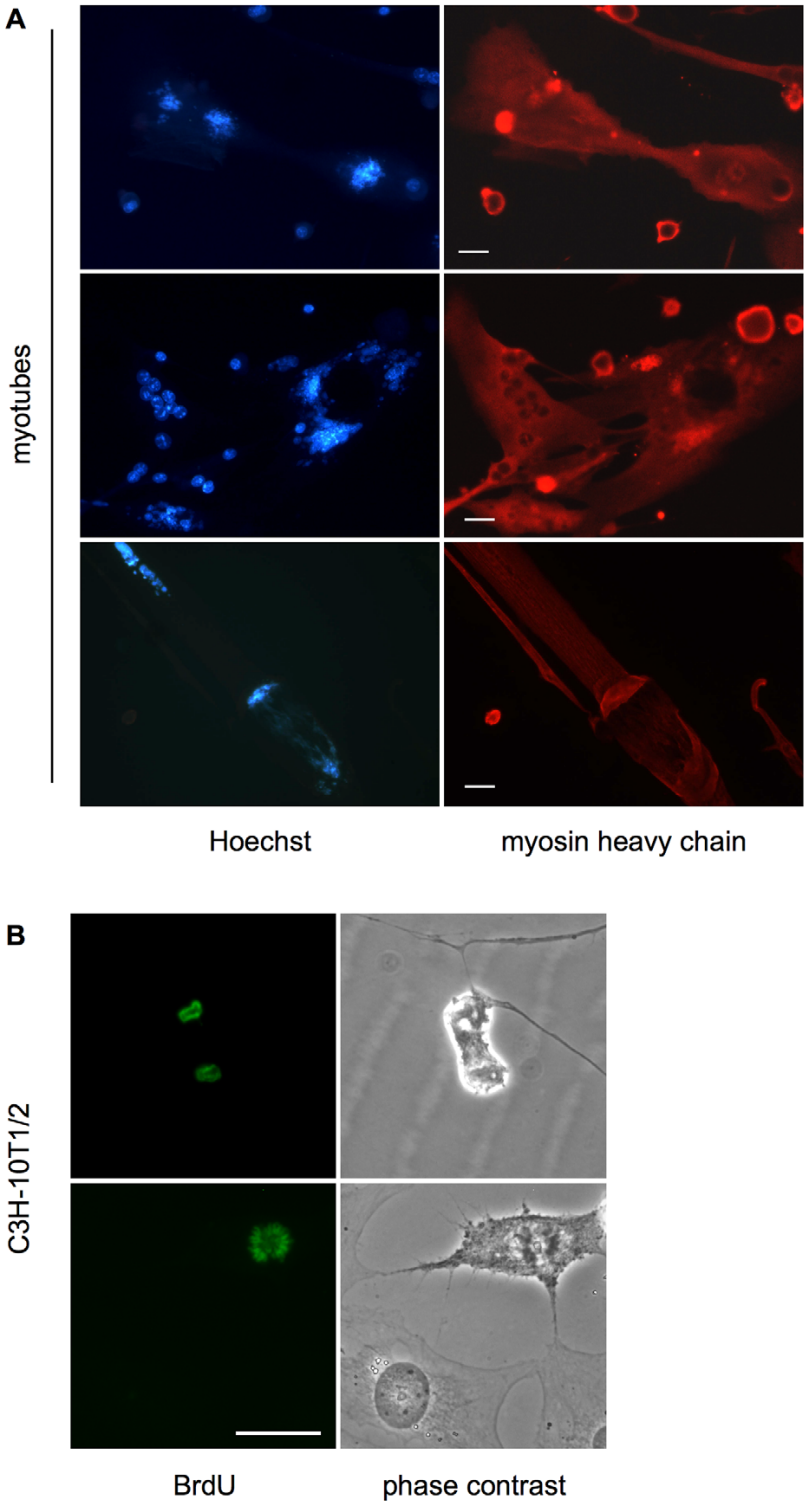
Aberrant mitoses in CKI KD-reactivated myotubes but not in fibroblasts. (**A**) MSC-derived myotubes were transfected with siRNAs to p21 and p27. The cells were fixed and stained 46 hours later. Bar  = 25 µm. (**B**) Serum-starved, quiescent C3H-10T1/2 cells were transfected with siRNA to p21 and p27 and kept in medium containing 0.1% FBS. The cells were fixed and stained 44 hours later. Bar  = 12.5 µm.

Microscopic observation showed that the mitoses occurring in the reactivated myotubes are invariably extremely aberrant. While some mitoses display a large number of anomalies such as chromosomal breaks, DNA bridges, and chromosome missegregation, most others show widely scattered and/or pulverized chromosomes. In addition, myotubes surviving the mitotic phase show an often extreme degree of micronucleation, the result of nuclear membrane reassembly around isolated chromosome fragments (Fig. 2A). On the contrary, quiescent fibroblasts reactivated by the same means undergo morphologically normal mitoses (Fig. 2B).

Differentiated muscle fibers and myotubes lack detectable centrosomes [21] and have atypical microtubule organizing centers at best [22]. Thus, it is conceivable that myotubes forced to reenter the cell cycle might form dysfunctional mitotic spindles, justifying at least in part the mitotic aberrations we observed. To determine whether reactivated myotubes possess centrosomes and can organize mitotic spindles, we stained myotubes subjected to p21 and p27 knockdown (KD) with antibodies to γ- and β-tubulin. Supplementary material Fig. S1A shows that centrosomes are readily stained by the γ-tubulin antibody in proliferating MSC. In undisturbed myotubes, the centrosomes are undetectable (data not shown), consistent with previous reports [21]. However, reactivated myotubes promptly regenerated centrosomes, which were positioned in the proximity of nuclei (supplementary material Fig. S1A). In addition, the reactivated myotubes formed mitotic spindles that, although often anomalous in shape, are clearly recognizable as such (supplementary material Fig. S1B). Thus, neither the absence of centrosomes nor the lack of mitotic spindles is the likely cause of the deep mitotic anomalies. It should be noted that similar mitotic aberrations are routinely observed in TD, mononucleated muscle cells (myocytes) reactivated by CKI KD (data not shown). Hence, the abnormal mitoses cannot be ascribed to the presence of multiple nuclei in the myotube cytoplasm.

### Reactivated myotubes, but not quiescent cells, suffer heavy DNA damage

**Figure 3:**
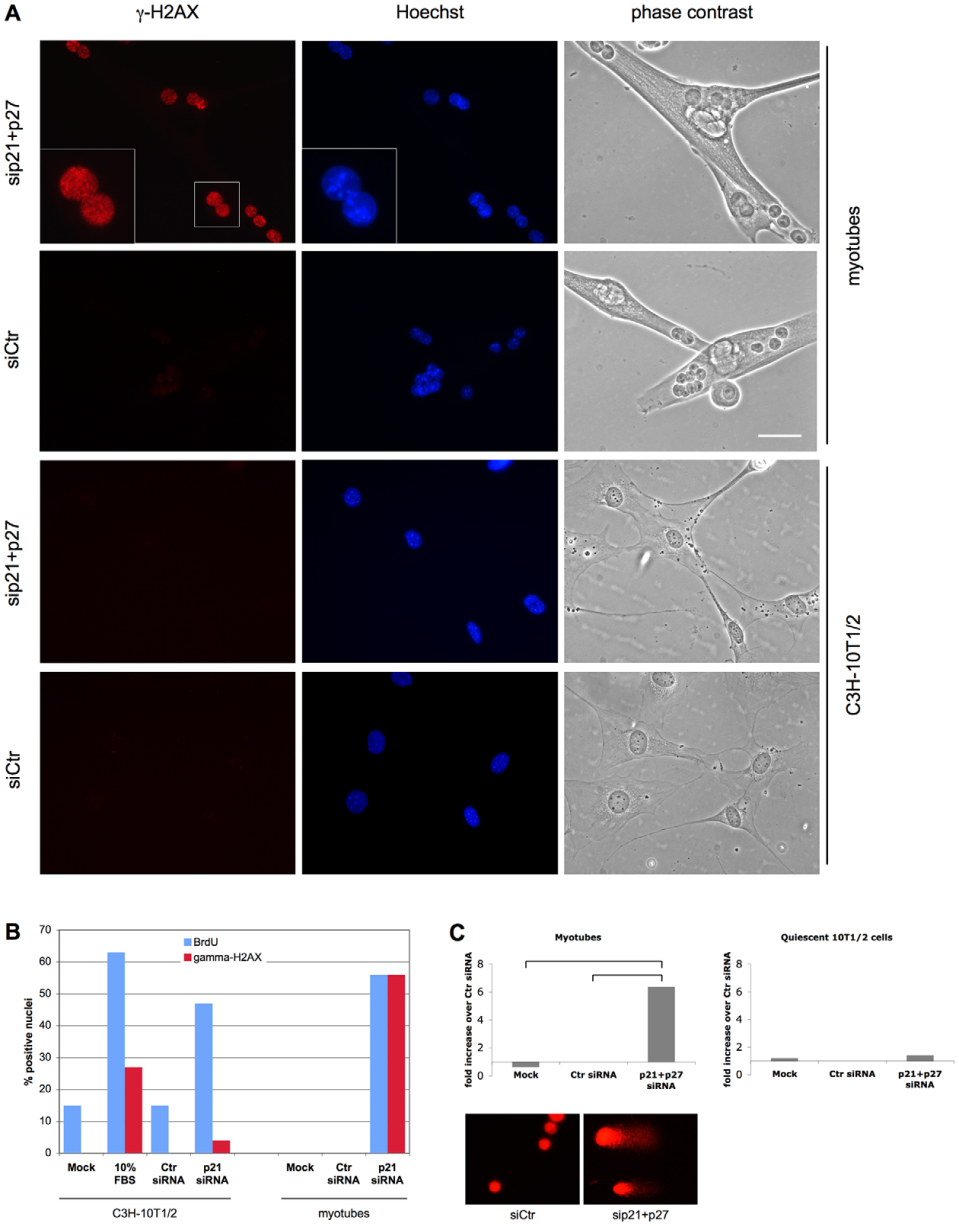
DNA damage in myotubes but not in fibroblasts reactivated by CKI KD. (**A**) MSC-derived myotubes and quiescent C3H-10T1/2 fibroblasts were transfected with the indicated siRNAs and stained 30 hours later. Bar  = 25 µm. The insets show magnifications of the area surrounded by a small, white square in the top left panel. (**B**) MSC-derived myotubes and quiescent C3H-10T1/2 fibroblasts were treated as indicated and double-stained for BrdU and γ-H2AX 48 hours later. The percentages of BrdU- and γ-H2AX-positive nuclei are shown. At least 100 nuclei per treatment were scored. (**C**) MSC-derived myotubes and quiescent C3H-10T1/2 fibroblasts were mock-treated or transfected with the indicated siRNAs and harvested 46 hours later. The cells were then embedded in agarose on microscope slides and lysed. The resulting free nuclei were subjected to electrophoresis, stained with ethidium bromide, photographed, and morphometrically evaluated. The histograms reflect the average tail moment of at least 50 comets per treatment. Cluster analysis and chi-square test showed the differences between histograms connected by brackets to be significant with p<0.001. No other differences are significant. The microfotographs show examples of comets derived from myotube nuclei treated as indicated. Ctr  =  control; siCtr  =  control, irrelevant siRNA; sip21+p27 =  mixture of equal amounts of siRNAs to p21 and p27.

The observation of innumerable breaks in the mitotic chromosomes of reactivated myotubes implies the presence of very high levels of DNA damage in the preceding interphase. To confirm this point, we stained p21/p27 KD cells with an antibody to Ser^139^-phosphorylated histone H2AX (γ-H2AX), a marker of double-strand DNA breaks. Fig. 3A,B illustrates that, while myotubes transfected with control siRNA showed only basal γ-H2AX staining, 100% of the BrdU-positive, reactivated muscle cells displayed an unusually intense staining for the marker. The staining was peculiar not only with regard to its intensity, but also to its pattern. Indeed, while the typical γ-H2AX staining pattern is represented by discrete nuclear foci [23], [24], corresponding to individual sites of damage, our myotubes displayed a confluent, almost homogeneous γ-H2AX distribution, suggesting the presence of very numerous DNA breaks. Conversely, serum-starved, quiescent C3H-10T1/2 fibroblasts, also reactivated by p21/p27 RNAi, showed no increase in γ-H2AX staining over the controls (Fig. 3A,B). It should be noted that, as previously reported [25], mitogen-mediated cell cycle reactivation (10% FBS for C3H-10T1/2 cells, Fig. 3B) induces a physiological increase in H2AX phosphorylation.

Notably, myotubes subjected to interference with either p21 or p27 alone showed neither DNA synthesis nor increased γ-H2AX staining (supplementary material Fig. S2). This evidence shows that ablation of each CKI per se does not bring about DNA damage and indicates that such damage is strictly connected with DNA replication which, in this system, requires double, p21/p27 KD.

A more direct way to assess DNA damage is the comet assay [26], where agarose-embedded nuclei are stained with ethidium bromide and subjected to electrophoresis to visualize DNA fragments that migrate away from intact DNA. Using this assay, we analyzed TD myotubes and quiescent fibroblasts transfected with p21/p27 siRNAs, control siRNA, or mock transfected. Reactivated myotubes displayed a high increase in DNA fragmentation, as expressed by the tail-moment parameter (see Materials and Methods), over control-transfected or unperturbed myotubes (Fig. 3C). In contrast, no significant tail moment increase was detected in fibroblasts after p21/p27 KD.

Altogether, these results show that reactivation causes heavy DNA damage in TD myotubes, but not in similarly treated quiescent fibroblasts. In turn, this implies that neither CKI suppression nor the ensuing cell cycle reactivation per se harms DNA, but that the observed damage must be rooted in the peculiar properties of TD myotubes.

### DNA synthesis in reactivated myotubes is incomplete

**Figure 4:**
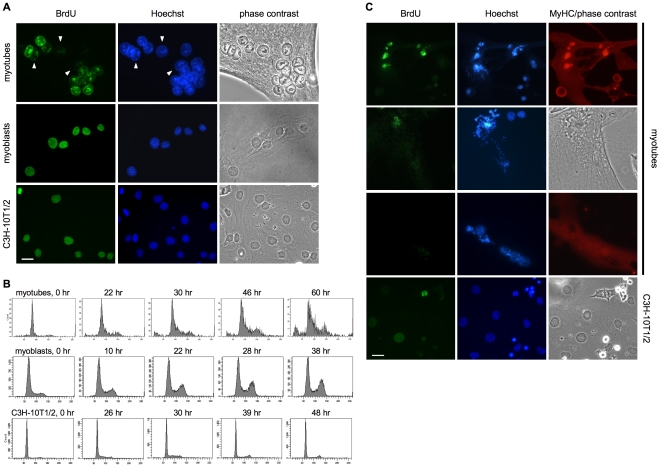
Incomplete DNA replication in myotubes but not fibroblasts reactivated by CKI KD. (**A**) MSC-derived myotubes and quiescent C2Q16 myoblasts and C3H-10T1/2 fibroblasts were transfected with siRNAs to p21 and p27 and continuously incubated with BrdU until fixation, 44 hours later. BrdU was detected by indirect immunofluorescence and nuclei were counterstained with Hoechst 33258. Arrowheads point at some incompletely, unevenly BrdU-substituted nuclei. Bar  = 12.5 µm. (**B**) C2Q16 myotubes and quiescent C2Q16 myoblasts and C3H-10T1/2 fibroblasts were transfected with siRNAs to p21 and p27, stained with propidium iodide, and analyzed by cytofluorimetry at the indicated time points. (**C**) MSC-derived myotubes and fibroblasts were treated and stained exactly as in (A). Mitotic figures were photographed. Bar  = 25 µm.

As described above, the chromosomal breaks observed in mitotic myotubes and the ensuing micronucleation strongly indicated the presence of DNA breaks. Another morphological characteristic of these mitoses, namely DNA bridges, suggested that DNA synthesis might be incomplete. Indeed, the BrdU staining pattern of myotube nuclei was irregular, even after very long BrdU labeling, suggesting that not all DNA regions are entirely duplicated (Fig. 4A). In contrast, quiescent C3H-10T1/2 fibroblasts and C2Q16 myoblasts reactivated by p21/p27 RNAi showed the expected homogeneous BrdU staining pattern (Fig. 4A).

To explore the completeness of DNA replication in myotubes, myoblasts, and fibroblasts reactivated as above, their nuclei were isolated and analyzed by cytofluorimetry. Fig. 4B shows that the majority of the reactivated myotube nuclei never reached 4n DNA content, while fibroblasts and myoblasts readily did so.

Further, striking evidence of incomplete DNA synthesis in reactivated myotubes came from the observation that a significant fraction of their mitotic chromosomes are only partially substituted with BrdU (Fig. 4C), as illustrated by the much smaller area stained by BrdU antibodies, compared with that highlighted by Hoechst 33258. Once again, such partial BrdU incorporation was never observed in reactivated fibroblasts (Fig. 4C).

These results allow us to conclude that DNA replication is incomplete in reactivated myotubes and that such partial duplication is specific to TD myotubes and does not take place in similarly reactivated non-TD cells. In addition, the occurrence of nuclei undergoing mitosis in the face of incomplete DNA replication implies a deep impairment of the G_2_ checkpoint.

Partial DNA replication might stem from defective reactivation of the cell cycle apparatus in TD myotubes. Although past investigations have shown that the cell cycle machinery can be reactivated in TD cells, much as it is in reversibly quiescent ones [17], [27], subtle functional anomalies might have escaped detection. We reasoned that, since aberrant DNA duplication is so prominent in TD cells, any such anomalies would be likely to directly involve components of the DNA replication apparatus or indirectly impinge on them. Thus, we investigated the expression of a number of factors involved in DNA replication in the reactivated myotubes. Np95 and cdc6 were found to be correctly reexpressed in these cells, at levels comparable to those of proliferating MSC, while cdc7 levels were shown to be independent of the replicative status of the cells (supplementary material Fig. S3). Mcm2, PCNA, and Ligase I were also properly expressed and associated with chromatin (supplementary material Fig. S3). In short, we were unable to reveal any TD cell-specific defects in the replication machinery. One possible interpretation of these negative results is that the obstacle to DNA replication might be structural (e.g., impervious chromatin structure), rather than functional.

### Reactivated myotubes undergo apoptosis

**Figure 5:**
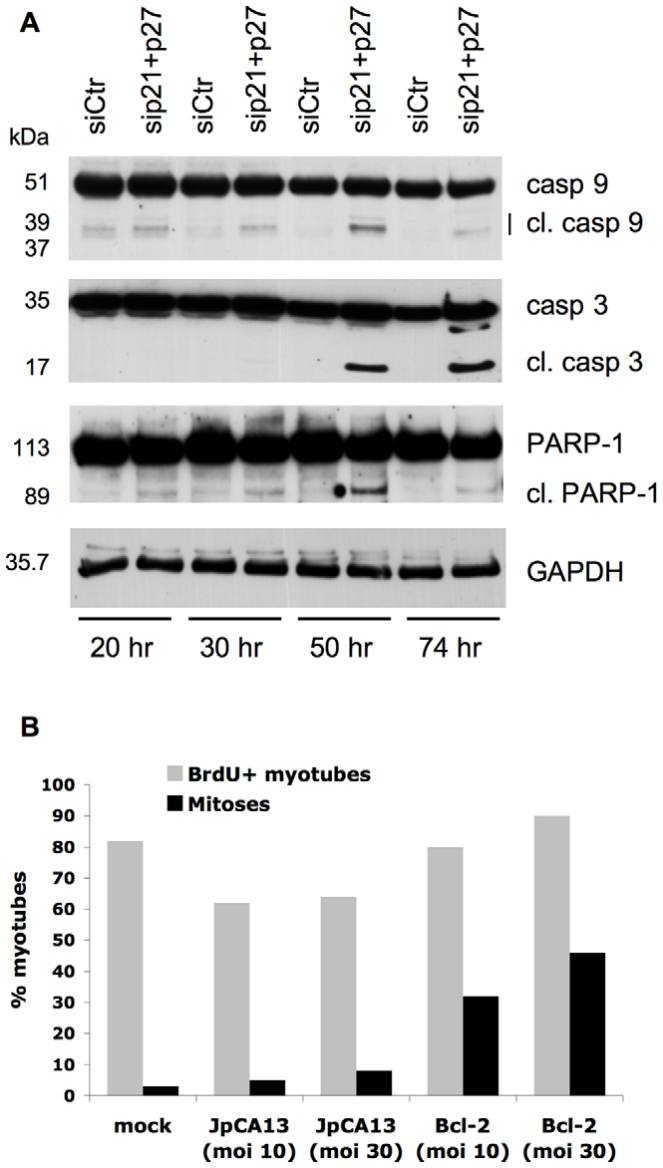
Myotubes reactivated by CKI KD undergo apoptosis, which can be delayed by forced Bcl-2 expression. (**A**) MSC-derived myotubes were transfected with the indicated siRNAs and harvested at the time points shown. The indicated proteins were analyzed by western blotting. GAPDH was used as a loading control. (**B**) MSC-derived myotubes were transfected with siRNAs to p21 and p27 and mock-treated (mock) or infected with an empty adenoviral vector or a Bcl-2-carrying virus at the indicated multiplicities of infection. The myotubes were continuously incubated with BrdU until fixation, 48 hours later. BrdU incorporation was detected by immunofluorescence and nuclei were counterstained with Hoechs 33258. The percentages of BrdU-positive (BrdU+) and mitotic or micronucleated myotubes (mitoses) are shown. Casp  =  caspase; cl.  =  cleaved; moi  =  multiplicity of infection.

As already described (Fig. 1), myotubes quickly die following cell cycle reactivation. To determine whether they die by apoptosis, we assessed cleavage-mediated activation of several molecules that regulate and effect apoptosis. As shown in Fig. 5A, caspase 3, caspase 9, and PARP-1 are all cleaved in a time-dependent fashion in reactivated myotubes, but not in control-transfected cells.

To delay cell death, we used a recombinant adenovirus to express the antiapoptotic gene Bcl-2 in MSC-derived myotubes. Bcl-2 expression did allow the reactivated myotubes to conserve a healthy morphology and survive much longer than those infected with a control adenovirus (not shown). Fig. 5B shows that Bcl-2 expression did not appreciably affect the proportion of myotubes undergoing DNA synthesis, but did allow a much higher percentage of them to enter mitosis, in a dose-dependent fashion. However, the mitoses of Bcl-2- and control-infected myotubes were not morphologically different, showing that suppression of apotosis does not relieve DNA damage.

Altogether, these results show that the reactivated myotubes die by apoptosis and that cell death is triggered early enough as to compete with entry into mitosis. Indeed, delaying programmed cell death allows a large fraction of myotubes that would otherwise die in the course of an unsuccessful S phase to reach mitosis.

### DNA damage is generated very early during S phase and is strictly associated with DNA replication

**Figure 6:**
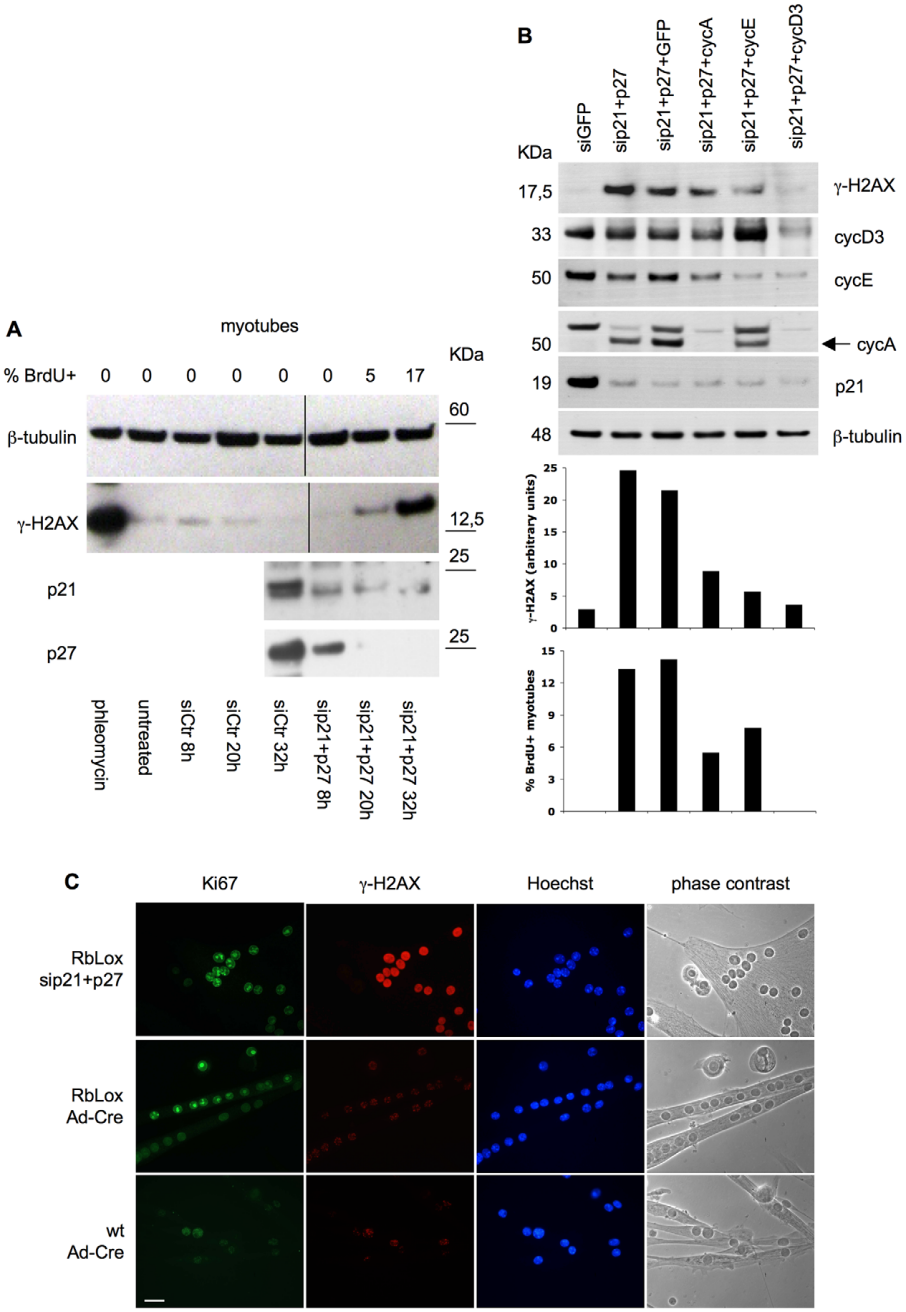
Histone H2AX phosphorylation in myotubes reactivated by CKI KD. (**A**) MSC-derived myotubes, transfected with the indicated siRNAs, were analyzed at the time points shown for BrdU incorporation and, by western blotting, for histone H2AX phosphorylation in serine 139, p21, and p27. Phleomycin treatment (50 µg/ml for 5 hours) was included as a positive control. β-tubulin is a loading control. Part of the β-tubulin, p21, and p27 western blots have been already shown in Fig. 1A. (**B**) MSC-derived myotubes, transfected with the indicated siRNAs, were harvested 42 hours after the transfection and analyzed for the proteins shown by western blotting. γ-H2AX abundance was quantitated by densitometry (upper graph); the percentages of BrdU-positive myotubes are shown in the lower graph. cyc  =  cyclin. (**C**) Wild-type (wt) or RbLox myotubes were transfected with siRNAs to p21 and p27 or infected with a Cre-carrying adenovirus (Ad-Cre). The cells were fixed 30 hours after transfection or 60 hours after infection and stained for the indicated proteins. Ki67 is a proliferation marker.

We wished to determine when DNA damage is engendered during the cell cycle in reactivated myotubes. For this purpose, γ-H2AX was analyzed in MSC-derived myotubes at various time points after p21/p27 siRNA transfection. Fig. 6A shows that γ-H2AX is already increased 20 hours after transfection, when only 5% of the myotubes have begun to synthesize DNA.

To further dissect the cell cycle and the timing of DNA damage in reactivated TD muscle cells, we knocked down, along with p21 and p27, the D3, E1, or A2 cyclin. Fig. 6B shows that in this setting DNA synthesis, as already reported for C2C12 myotubes, critically depends on cyclin D3. In contrast, KD of either cyclin E1 or cyclin A2 only reduced the percentage of myotubes undergoing DNA synthesis. Interestingly, the amount of DNA damage, as estimated from γ-H2AX levels, paralleled the number of myotubes entering S phase. These results suggest that the DNA damage arising in reactivated myotubes is strictly associated with DNA synthesis.

Since DNA damage occurs so early upon entry into S phase (Fig. 6A), conceivably it might even shortly precede it. To try and clarify this point, we used myotubes derived from Rb conditional knockout (RbLox) MSC. As previously described [27], when the Rb gene is acutely deleted through Cre recombinase expression, these myotubes activate a variety of molecular events that usually precede and associate with transition into S phase, including transcription of E2F target genes, increases in cyclin-E and –A associated kinase activities, enhanced expression of PCNA, DNA ligase I, RPA, and MCM2. However, though poised to undergo DNA duplication, these myotubes do not synthesize DNA. Fig. 6C shows that Rb-LoxP myotubes expressed Ki67 in response to both p21/p27 KD and Cre-mediated Rb KD. However, only the former treatment brought about an increase in γ-H2AX. Exactly as reported in Fig. 3, 100% of the myotubes reactivated by p21/p27 KD (n≥100) were stained heavily for γ-H2AX, while all of the Ad-Cre-infected controls showed only background staining (Fig. 6C). Thus, these experiments strongly indicate that DNA damage depends on DNA synthesis, rather than any preceding events.

### DNA damage follows DNA synthesis in myotubes, irrespective of the cell cycle reactivation mechanism

**Figure 7:**
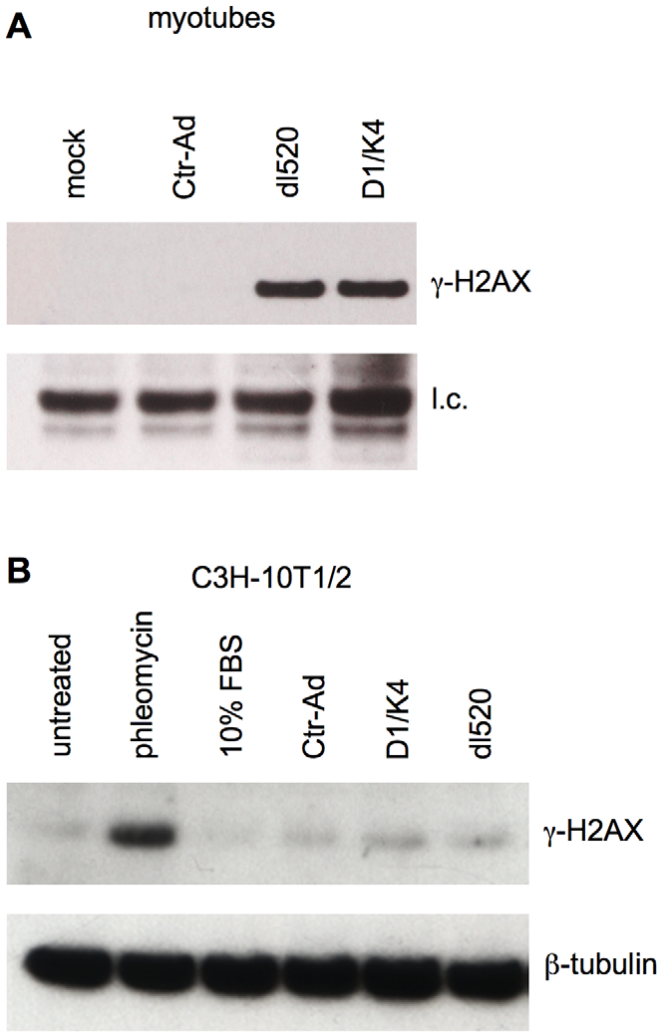
Phosphorylation of histone H2AX in myotubes but not fibroblasts reactivated by different strategies. C2C12 myotubes and quiescent C3H-10T1/2 fibroblasts were infected with cell cycle-reactivating viruses expressing either E1A (dl520) or cyclin D1 and cdk4 (D1/K4). The cells were also infected with an empty control virus (Ctr-Ad) or mock-infected. In addition, fibroblasts were reactivated with 10% FBS or treated with Phleomycin to provide a positive control for DNA damage. γ-H2AX was analyzed by western blotting. Multiplicities of infection as follows. Myotubes: Ctr-Ad 400, dl520 200, cyclin D1 60, cdk4 760. C3H-10T1/2: Ctr-Ad 300, cyclin D1 30, cdk4 300, dl520 200. l.c.  =  loading control.

Conceivably, the DNA damage observed in reactivated myotubes might be linked to the specific means used to trigger cell cycle reentry. To exclude this possibility, we reactivated both myotubes and quiescent fibroblasts by infection with the mutant adenovirus, dl520 [17], or expression of cyclin D1 and cdk4 [9]. As shown in Fig. 7, myotubes reactivated in either way displayed strong increases in γ-H2AX levels, while fibroblasts brought into the cell cycle by the same means did not.

Thus, we conclude that TD myotubes possess intrinsic features that prevent full DNA replication and cause damage as a consequence of attempted DNA synthesis.

## Discussion

Many reports in the literature describe a variety of approaches to the problem of reactivating the cell cycle in TD cells. However, the final fate of the reactivated cells is less often described or investigated.

Here we show that TD skeletal muscle cells subjected to CKI RNAi do reenter the cell cycle, but cannot complete DNA replication, suffer severe DNA damage, and die by apoptosis or as a consequence of profoundly disrupted mitoses. In striking contrast, reversibly quiescent fibroblasts reactivated in the same way fully replicate their DNA, suffer no detectable damage, and in fact proliferate even in the absence of exogenous growth factors. We have previously shown that even permanently non-dividing, senescent cells can be induced to replicate and proliferate by CKI KD with no induced cell death [10]. These observations suggest that TD myotubes are characterized by intrinsic attributes that prevent proper DNA replication, even when induced by means that are effective in non-TD cells.

In TD myotubes, entry into S phase is immediately followed by accumulation of DNA damage, as best shown by significant increases in γ-H2AX early after CKI KD, when only a small minority of the myotubes have entered S-phase (Fig. 6A). The incompleteness of DNA replication in myotubes is another remarkable feature of cell cycle reactivation in these cells, shown by cytofluorimetric measurements of DNA content and partial BrdU substitution in mitotic chromosomes. To our knowledge, incomplete DNA replication as a characterizing feature of cell cycle reactivation in TD cells has not been previously described. The likely occurrence of DNA damage in reactivated TD cells has been reported before, as activation of the DNA damage response (DDR) [14], [16]. Here, using the comet assay, we show directly DNA fragmentation and correlate it with incomplete DNA synthesis.

At the present time, it is unclear whether DNA damage is the cause or the effect of partial DNA replication. On one hand, it is possible that chromosomal damage, possibly resulting from attempted replication in an unpermissive cellular environment, could bring about S-phase checkpoint activation and consequent slow-down/arrest of DNA replication [28], [29]. Alternatively, slow DNA replication might be intrinsic to myotubes, due for example to unique chromatin organization, and the attempt to push replication through structural obstacles might result in DNA damage.

Irrespective of its primary cause, DNA damage triggers apoptosis, to which most myotubes succumb. Some of the cells, however, enter M phase in spite of incomplete DNA replication and heavy chromosomal damage, both of which should activate the G_2_ checkpoint and prevent the onset of mitosis. However, the reactivated myotubes have been depleted of p21, which has been shown to be required for sustained G_2_ arrest [30]. Thus, myotubes surviving apoptosis long enough enter mitosis with fragmented and incompletely replicated DNA, resulting in mitotic catastrophe. Accordingly, prevention of apoptosis by expression of Bcl-2 brings a far higher number of myotubes into mitosis (Fig. 5B).

A review of the literature concerning reactivation of the cell cycle in a variety of TD cells shows that, in most or possibly all instances, such cells undergo either death or indefinite growth arrest. In particular, cell death follows reactivation of myotubes with Large T antigen [20], E1A [31], or CKI KD. Inner-ear cells triggered to reenter the cell cycle by HPV E7 expression also die [16]. Examples of indefinite proliferation arrest in G2 phase include cardiomyocytes reactivated by the expression of E1A, E2F1 [12], or Notch2 intracellular domain [14]. Interestingly, although myotubes die in response to, e.g., CKI KD, they arrest in G2 phase when reactivated by overexpression of cyclin D1 and cdk4 [9]. This indicates that the specific outcome of reactivation is dictated not only by the cell type considered, but also by the method used to achieve cell cycle reentry.

We propose a unifying model, applicable to most TD cells, that reconciles these two apparently divergent outcomes. We submit that a wide variety of TD cells are intrinsically unable to effect complete DNA replication. Thus, attempts to force these cells to divide bring about extensive DNA damage, which can determine different, non mutually exclusive end results: programmed cell death as a result of DDR activation, arrest in G_2_ phase if the corresponding checkpoint is functional, or entry into a catastrophic mitosis if it is not. Likely, cell-type specific variations on this mechanism exist. For example, TD cardiomyocytes physiologically allow a single round of DNA replication not followed by cell division [32]. Thus, cardiac cells might have strong mechanisms preventing them from entering mitosis even after forced DNA replication, whence their preference for arresting in G_2_ phase [12], [14]. We believe that the virtue of the proposed model lies in its focusing future efforts onto finding the specific, critical feature(s) of TD cells, whether structural or functional, that causes DNA damage and/or hinders completion of DNA replication upon cell cycle reentry.

Our observations might help explain why mammalian TD cells, e.g., myotubes, unlike homologous cells in regenerating vertebrates, e.g., urodele amphibians, cannot contribute to tissue repair [2]. Although muscle cells from both groups of organisms can be reactivated, only those from regenerating species yield viable progeny that help reconstitute tissue integrity [33].

We do not exclude the possibility that future methods might attain full, harmless reactivation of TD cells. Indeed, our efforts focus on the discovery of such methods. However, we would like to underscore that the dramatically different responses of TD vs. non-TD cells to the same proliferation-activating treatments point at a hitherto poorly recognized uniqueness of TD cells and indicate that the TD state is intrinsically—if not absolutely—incompatible with DNA replication. We point out that there is no current biological explanation for the observed phenomena, indicating that they are grounded in uncharted territory.

## Materials and Methods

### Cells and adenoviruses

Primary MSCs were isolated and cultured as previously described [34]. Differentiation was induced by plating the cells on gelatin-coated dishes (Iwaki, Tokio, Japan) and culturing them in DMEM supplemented with 10% foetal bovine serum (FBS) for 72 hours. Rb^LoxP/LoxP^ (RbLox) MSC have been described [27]. These cells were cultured exactly like wild-type MSC. The mouse C2C12 myoblast cell line [35] was cultured as previously described [9]. Differentiation was induced by plating the cells in collagen-coated dishes (Iwaki) and culturing them in DMEM supplemented with 5 ng/ml human insulin, 5 ng/ml human holo-transferrin and 1 mM dexamethasone for three days. To eliminate undifferentiated cells, 50 mM cytosine-D-arabinofuranoside was added for the first 48 hours only where stated. C2Q16 cells are a previously described subclone of C2C12 cells [36], characterized by slower but more efficient differentiation, compared to the parental cells. C2Q16 cells were cultured and induced to differentiate exactly like C2C12 myoblasts. Murine C3H-10T1/2 fibroblasts were cultured in DMEM supplemented with 10% FBS and brought into quiescence by culturing them for at least 48 hours in DMEM containing 0.1% FBS.

The dl520 virus is a deletion mutant of type 5 human adenovirus expressing 12S, but not 13S, E1A [9]. This virus was used at multiplicity of infection (MOI) 70, 150, or 200. The Bcl-2 recombinant adenovirus was generated using the Ad-Easy system [37]. Briefly, a human Bcl-2 cDNA was cloned into the pAd-track-CMV shuttle vector. This vector and the pAd-Easy backbone were cotransfected into 293 cells and the resulting recombinant viruses were plaque-purifed and amplified in 293 cells. Overexpression of human Bcl-2 was obtained by infecting myotubes at MOI 10 or 30. The E1-deleted J-pCA13 virus [38] was used as a negative control.

### RNAi experiments and siRNAs

Downregulation of target genes in C2C12 and MSC myotubes was achieved by RNAi as already described [10]. Briefly, the HiPerFect reagent (QIAGEN, Hilden, Germany) was used to complex single or multiple siRNAs and transfection complexes were kept in the culture medium (DMEM supplemented with 10% or 5% FBS for MSCs or C2C12, respectively) until the end of the experiments. When requested, adenoviral infection was performed 1 hours before the RNAi procedure.

siGenome individual duplex siRNAs (Dharmacon, Colorado, USA) were used to interfere with the following mouse transcripts: CDKN1A (p21, 5′-GAACAGGUCGGACAUCACCUU); CCND3 (cyclin D3, 5′-AAUCACGGCAGCCAGGUCCUU); CDKN1B (p27, 5′-UAUCCCGGCAGUGCUUCUCUU). siGenome SMARTpool siRNA reagents (Dharmacon) were used to interfere with DNA polymerase α RNA. SASI duplex siRNAs (Sigma-Aldrich, St. Louis, MO, USA) were used to interfere with murine cyclin A (5′-AAUACUAGGUGCUCCAUUCdTdT) and cyclin E (5-AGCCAAUCCAGAAGAACUGdTdT). Non-Targeting siRNA #2 (Dharmacon) or duplex siRNA to green fluorescent protein (5′-GGCUACGUCCAGGAGCGCACC, MWG, Ebersberg, Germany) were transfected as controls.

### Immunofluorescence and cytofluorimetry

For immunofluorescence analysis, cells were fixed with 2% formaldehyde and permeabilized in 0.25% Triton X-100/PBS. The following monoclonal antibodies (mAbs) were used. BrdU: Bu20a clone (DAKO, Glostrup, Denmark); myosin heavy chain: MF20 [39]; γ-H2AX (cat. 07-164; Millipore, Bllerica, MA, USA). AlexaFluor-conjugated antisera to mouse IgG were obtained from Invitrogen (San Diego, CA, USA). Nuclei were counterstained with Hoechst 33258.

Cell cycle analyses was performed with nuclei isolated from MSC-derived myotubes. Myotubes were incubated in 10 mM Hepes, pH 7, 10 mM KCl, 0.1 mM EDTA, 0.1 mM EGTA for 1 hour on ice. NP-40 was added at a final concentration of 0.6% and the cell suspension was vigorously vortexed for 30 seconds. After a brief centrifugation, the nuclear pellet was washed with PBS, fixed in methanol:acetone 4∶1, and stained in 50 mg/ml propidium iodide, 200 mg/ml RNAse A, 0.2% Tween 20. An FACS Canto cytofluorimeter (Becton Dickinson) was used to analyze the samples.

### Single cell gel electrophoresis (comet assay)

Alkaline single cell gel electrophoresis was performed as described [40]. Briefly, cell suspensions were mixed with 0,5% low melting point agarose/PBS (BioRad, Hercules, CA, USA) and applied to glass microscope slides coated with 1% low melting point agarose. The gel was allowed to solidify at 4°C, then the slides were submerged in lysis solution (2.5 M NaCl, 100 mM EDTA, 10 mM Tris, 1% Triton X-100 and 10% DMSO, pH 10) and kept at 4°C over night. The slides were then incubated for 20 minutes in alkaline buffer (300 mM NaOH, 1 mM EDTA, pH 13). Electrophoresis was performed in alkaline buffer at 300 mA for 30 minutes at room temperature. After electrophoresis, the slides were placed in neutralization buffer (0.4 M Tris-NaOH, pH 7.5) for 5 minutes and then fixed in cold 100% ethanol for 5 minutes. Slides were air dried and stained with ethidium bromide, 20 µg/ml in water.

Comet images were analyzed using the IAS 2000 7.0 software (Delta Sistemi, Roma, Italy). At least 50 nuclei per experimental point were analyzed. The tail moment parameter is calculated as (percentage DNA in the tail) × (head-to-tail distance). Nuclei with apoptotic morphology (very small comet head and extremely large tail) were excluded from analysis.

### Total cell extracts, chromatin isolation, and Western blots

To prepare total cell extracts, cells were harvested, washed in PBS, and lysed with RIPA buffer. Chromatin extracts were prepared following a previously published method [41], with minor modifications. Briefly, cells were resuspended in buffer A (10 mM Hepes, pH 7.9, 10 mM KCl, 1.5 mM MgCl_2_, 0.34 M sucrose, 10% glycerol, 0.5 mM EGTA, 0.1% Triton X-100, 0.5 mM PMSF, 1 mM DTT, 1 mM ATP, protease inhibitor cocktail (Roche, Basel, Switzerland) and incubated 10 minutes on ice. Nuclear pellet 1 (P1) and supernatant 1 (S1) were separated by low-speed centrifugation. S1 was further centrifuged at high speed to separate cytoplasmic soluble proteins (S2) from debris (P2). P1 nuclei were washed once with buffer A and then lysed in buffer B (3 mM EDTA, 0.5 mM EGTA, 0.5 mM PMSF, 1 mM DTT, 1 mM ATP, protease inhibitor cocktail) for 30 minutes on ice. Nuclear soluble proteins (S3) and insoluble chromatin-bound proteins (P3) were separated by centrifugation. S2, S3, and P3 fractions were resuspended in Laemmli buffer and briefly sonicated.

Proteins were separated on gradient, 4–12% polyacrylamide gels and analyzed by western blotting with the following mAbs or polyclonal antibodies (pAbs): mAbs to γ-H2AX (Millipore), PARP-1 (Becton Dickinson, Franklin Lakes, NJ, USA), caspase-9 (Cell Signaling, Danvers, MA, USA), cyclin D1, GAPDH, cdc6, PCNA (PC10) from Santa Cruz Biotechnology, Santa Cruz, CA, USA; p27 (610241) from Becton Dickinson Transduction Laboratories); β-tubulin (Sigma-Aldrich), NP-95 (a kind gift of I.M. Bonapace), and Ligase 1 [42]; pAbs to mouse Apaf-1 (Millipore), caspase-3 (Cell Signaling), cdk4 (Ab-5) from NeoMarkers, Thermo, Waltham, MA, USA; cdc7, p21, cyclin D3, cdk2, cdk6, cyclin A, cyclin E, and MCM-2 (sc-9839) from Santa Cruz Biotechnology. Peroxidase-conjugated antisera to mouse and rabbit IgG were from Bio-Rad Laboratories. Western blots were revealed using the SuperSignal kit (Pierce Chemical Co., Rockford, IL, USA).

## Supporting Information

Figure S1 Centrosome and mitotic spindle detection in reactivated miotubes. MSC-derived myotubes reactivated by CKI KD and proliferating MSC were immunostained for the centrosome marker γ-tubulin (A) or the microtubule constituent β-tubulin (B). Nuclei were counterstained with Hoechst 33258.
(1.26 MB TIF)

Figure S2 Single-CKI KD does not elicit DNA damage. MSC-derived myotubes were transfected with siRNAs to p21, p27, or both. The cells were fixed 30 hours later, immunostained for the indicated proteins, and countestained with Hoechst 33258.
(1.01 MB TIF)

Figure S3 DNA replication machinery in CKI KD-reactivated myotubes. (A) MSC-derived myotubes were transfected or infected as shown and the indicated proteins were analyzed by western blotting at successive time points. Proliferating MSC are included for reference. (B) MSC-derived myotubes were transfected as shown and the indicated proteins were analyzed by western blotting at successive time points. the graph reports the percentages of BrdU-positive cells at successive time points. (C) MSC-derived myotubes were transfected as indicated and compared with quiescent or serum-stimulated C3H-10T1/2 cells. Total cell extracts were fractionated into soluble cytoplasmic (S2), soluble nuclear (S3), and chromatin (P3) fractions and analyzed for the indicated proteins by western blotting 30 hours later. Whole cell lysates (WCL) from myotubes (Mt) and MSC are included for reference. The percentages of BrdU-positive cells are shown in the bottom table.
(1.36 MB TIF)
