# Supplementary figures and images for: DNA Replication Is Intrinsically Hindered in Terminally Differentiated Myotubes
Source: PLoS One. 2010 Jul 13;5(7):e11559. doi: 10.1371/journal.pone.0011559 (PMC2903488; doi:10.1371/journal.pone.0011559)

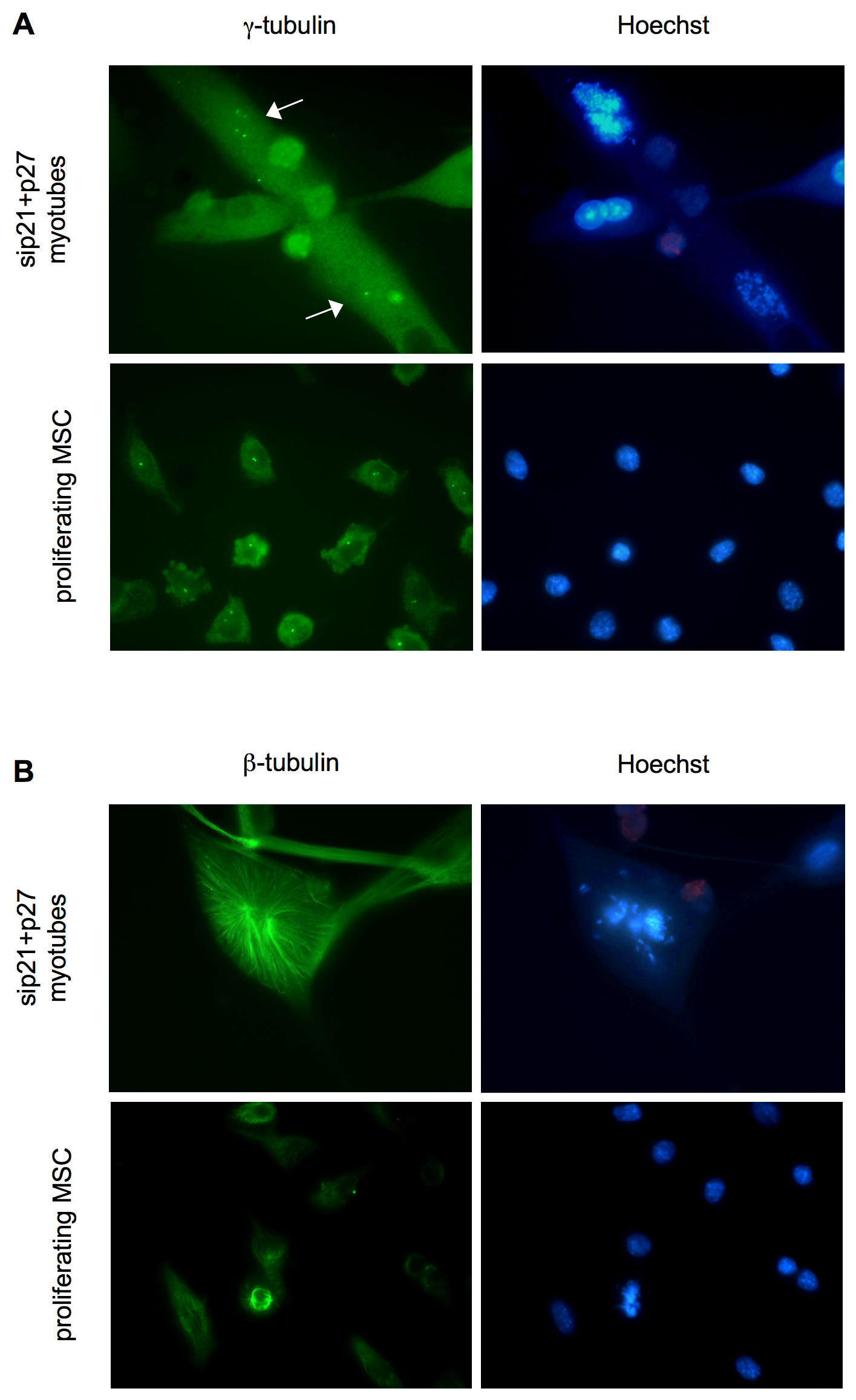

Supplement: Figure S1 — Centrosome and mitotic spindle detection in reactivated miotubes. MSC-derived myotubes reactivated by CKI KD and proliferating MSC were immunostained for the centrosome marker γ-tubulin (A) or the microtubule constituent β-tubulin (B). Nuclei were counterstained with Hoechst 33258. (1.26 MB TIF) [file pone.0011559.s001.tif]

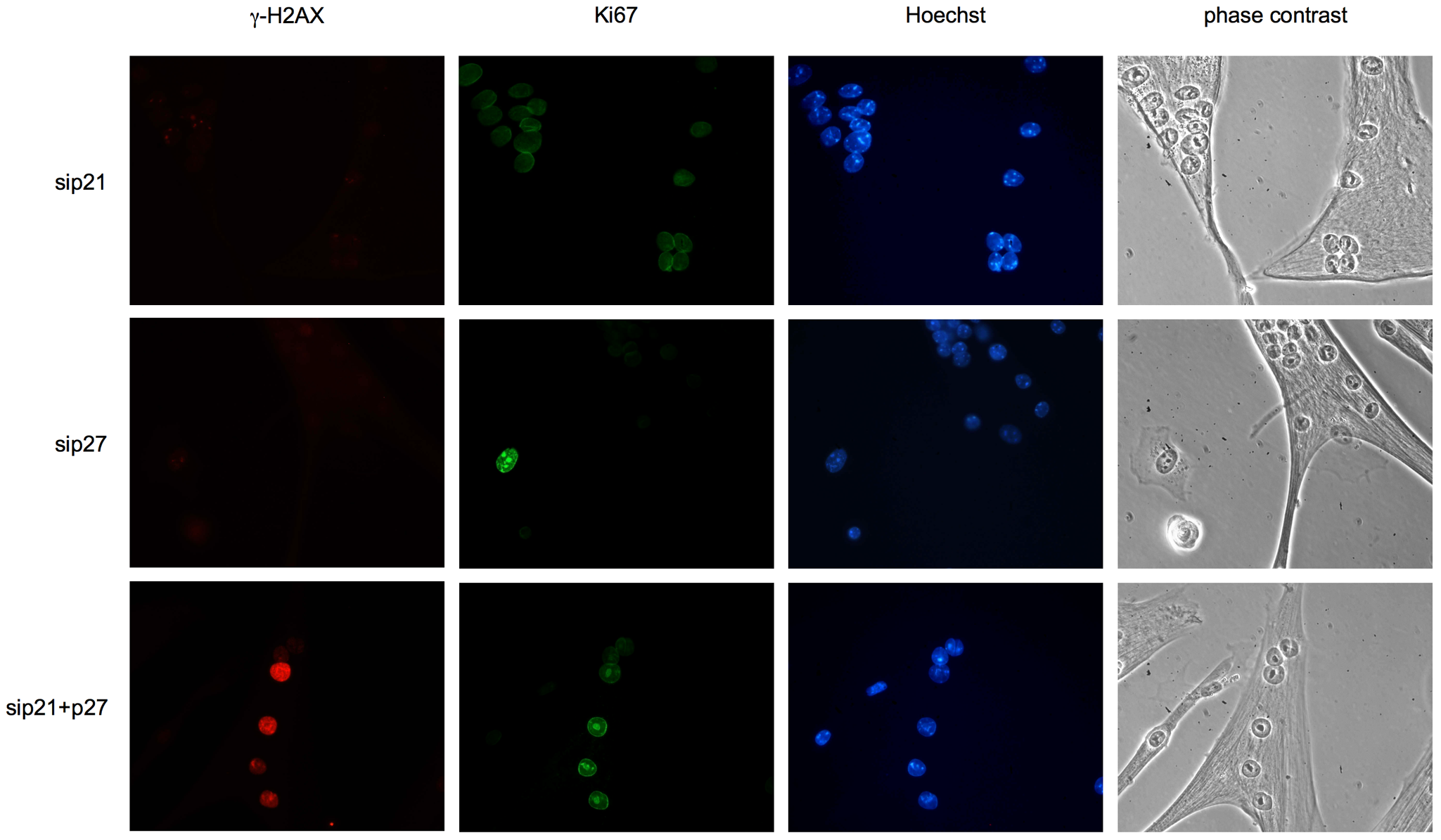

Supplement: Figure S2 — Single-CKI KD does not elicit DNA damage. MSC-derived myotubes were transfected with siRNAs to p21, p27, or both. The cells were fixed 30 hours later, immunostained for the indicated proteins, and countestained with Hoechst 33258. (1.01 MB TIF) [file pone.0011559.s002.tif]

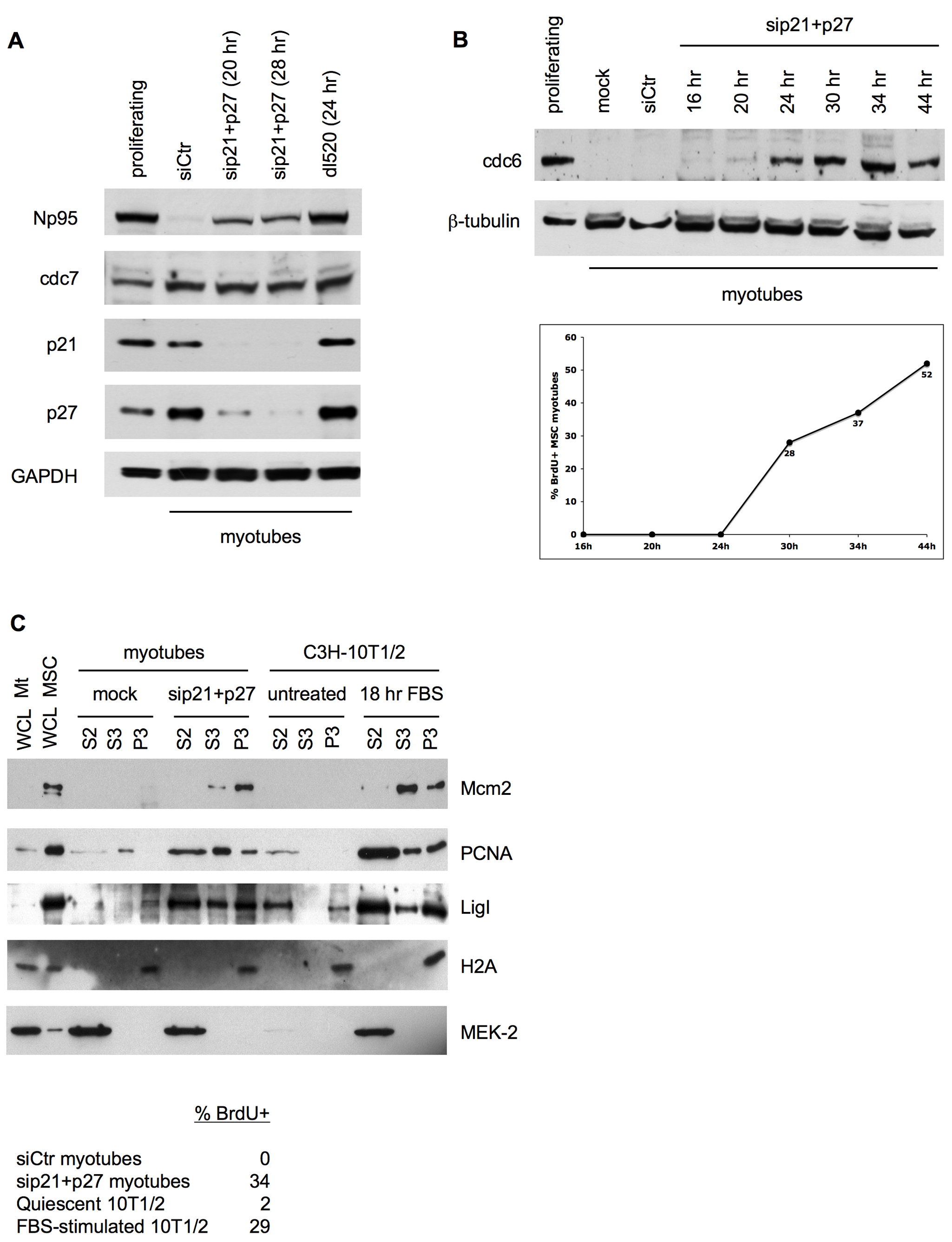

Supplement: Figure S3 — DNA replication machinery in CKI KD-reactivated myotubes. (A) MSC-derived myotubes were transfected or infected as shown and the indicated proteins were analyzed by western blotting at successive time points. Proliferating MSC are included for reference. (B) MSC-derived myotubes were transfected as shown and the indicated proteins were analyzed by western blotting at successive time points. the graph reports the percentages of BrdU-positive cells at successive time points. (C) MSC-derived myotubes were transfected as indicated and compared with quiescent or serum-stimulated C3H-10T1/2 cells. Total cell extracts were fractionated into soluble cytoplasmic (S2), soluble nuclear (S3), and chromatin (P3) fractions and analyzed for the indicated proteins by western blotting 30 hours later. Whole cell lysates (WCL) from myotubes (Mt) and MSC are included for reference. The percentages of BrdU-positive cells are shown in the bottom table. (1.36 MB TIF) [file pone.0011559.s003.tif]
